# Obituary

**Published:** 1905-04-15

**Authors:** 


### OBITUARY.

DR. JAMES T. IRWIN, for many years a prominent practitioner of dentistry in Cincinnati, died February 21st, 1905, at his home in Cincinnati, at the age of 72 years. He graduated from the Ohio College of Dental Surgery in 1853. He was associated in practice at one time with Dr. James Taylor, with whom he studied dentistry. He was a well-known practitioner in his own city, but as he never engaged in professional literature or scientific work he was not known outside of Cincinnati, where he practiced. He retired from practice several years before his death.

DR. JAMES LESLIE, of Cincinnati, died February 8th, 1905, at his home in Cincinnati, at the age of 87 years. Dr.

Leslie was never a practitioner of dentistry, but was for a long time interested in the practice of dentistry from the fact that he for many years was a manufacturer of gold foil for dentists and he had a brother who was a dentist. Dr. Leslie for many years made crystal gold and claimed that he was the first to put a usable product on the market. He and his brother were prominent in the west as dealers in dentists' supplies in Cincinnati and St. Louis. In 1892 the Ohio College of Dental Surgery conferred on him the degree of Doctor of Dental Surgery. He was one of the founders of the Ohio Dental College and for many years served on its board of trustees in various official capacities. He was a member of the old Mississippi Valley Dental Association, the first dental society ever organized, and was generally present at its meetings and took active part in its deliberations. His great interest was in gold as a filling material. He made a beautiful preparation of crystal gold, but it never became popular, because he did not push it in a business way as some other manufacturers did. His greatest hobby was the Ohio Mechanics' Institute which he helped to organize and in which he was greatly interested up to the time of his death.

